# Application of Transtheoretical Model on Behavioral Changes, and Amount of Physical Activity Among University’s Students

**DOI:** 10.3389/fpsyg.2018.02402

**Published:** 2018-12-17

**Authors:** Kien Ting Liu, Yee Cheng Kueh, Wan Nor Arifin, Youngho Kim, Garry Kuan

**Affiliations:** ^1^Unit of Biostatistics and Research Methodology, School of Medical Sciences, Universiti Sains Malaysia, Malaysia, Kelantan, Malaysia; ^2^Department of Sports and Health Science, Seoul National University of Science and Technology, Seoul, South Korea; ^3^Exercise and Sports Science, School of Health Sciences, Universiti Sains Malaysia, Malaysia, Kelantan, Malaysia

**Keywords:** decisional balance, processes of change, physical activity, structural equation modeling, self-efficacy, transtheoretical model

## Abstract

This study’s purpose was to examine the structural relationship of the transtheoretical model (TTM) and the amount of physical activity (PA) among undergraduate students in health and medicine at Universiti Sains Malaysia. A cross-sectional study was carried out among students who took part in the co-curricular program. Co-curricular program includes activities that take place outside of the regular lectures or tutorials in the University. Students recruited through purposive sampling were informed that their participation was entirely voluntarily. Those interested completed the self-administered questionnaire, which consisted of the decisional balance, processes of change, self-efficacy, stages of change scales, and Godin leisure-time exercise questionnaire. Data were analyzed using Mplus version 8 for descriptive statistics and structural equation modeling analysis for inferential statistics. A total of 562 students participated in the study. The majority of the students was female (79.0%) and Malay (73.3%) and average of exercise sessions per week was 2.62, with a mean of 43.37 min per exercise session. The final structural model fit the data well based on several fit indices (SRMR = 0.046, RMSEA (CI: 90%) = 0.061 (0.045, 0.078), RMSEA *p* = 0.130). The model showed that stages of change significantly affected self-efficacy (*p* < 0.001), pros (benefits of exercise; *p* < 0.001), cons (barriers to exercise; *p* = 0.022), and processes of change (*p* < 0.001). The model also showed significant inter-relationships among the TTM constructs and supported seven hypotheses. Among all the variables examined, only processes of change significantly affected PA (*p* < 0.001). However, stages of change (*p* < 0.001) and pros (*p* =< 0.001) had significant indirect effects on PA via processes of change. The findings support that individuals’ stages of change affect their self-efficacy level, or the ability to make positive and negative decisions and perform behavior accordingly. The study confirms that making correct decisions and taking action accordingly can increase PA levels.

## Introduction

Physical inactivity and sedentary behavior are considered to be global issues. Although numerous studies have highlighted the importance of physical activity (PA) in the general population (e.g., Mozaffarian et al., 2012), university and college students have been reported to have relatively low PA levels (U.S. Department of Health and Human Services, 2008; World Health Organization, 2010). Over the years, strategies have been carried out to increase PA levels, yet 20% of adults remain physically inactive (U.S. Department of Health and Human Services and U.S. Department of Agriculture, 2015). In Malaysia, most students aged 22–25 years have been reported to have low and moderate levels of PA (Rajappan et al., 2015). Malaysian university students have also been shown to engage in PA and exercise in at least once a week, with at least 30 min per session (Kueh et al., 2017). Numerous interventions to increase PA have been conducted among adults, yet not many have participated in PA or maintained PA in the long term (Institute for Public Health, 2014). To gain a better understanding of the psychological mechanisms involving students’ physical behavior, a theory-based intervention was developed to promote PA. PA is a complex behavior to modify and adopt (Rhodes and Nigg, 2011), but relevant interventions could benefit from the developed theory.

The transtheoretical model (TTM) is aimed at understanding individuals’ behavioral changes (Prochaska and DiClemente, 1983) and describing how people move dynamically through five different stages of behavioral changes. The TTM has been widely used to describe and understand exercise behavior, such as adoption and maintenance of PA (Han et al., 2017). The four core constructs of TTM are stages of change, self-efficacy, decisional balance, and processes of change. The TTM holds that people begin to perceive more benefits than disadvantages from adopting positive behavior changes as they move through the later stages (Han et al., 2015). This view was also supported by Prochaska and Velicer (1997), who stated that the cons outweigh the pros in the earlier pre-contemplation stage of change. In this study, the pros and cons in decisional balance construct refers to the benefit that encourage people to exercise and barriers that refrain people to exercise.

The transtheoretical model which act as central guideline to positive health-behavior changes, indicated that individuals who were attempting to change their health behavior might experience a series of stages of readiness for change, namely: precontemplation (not intending to make changes or denying the need for change), contemplation (seriously considering making changes), preparation (making small changes), action (actively engaging in exercise for less than 6 months), and maintenance (exercising regularly for at least 6 months) (Lee et al., 2006). Movement through these stages often occurs in cyclic rather than linear patterns because many individuals must make several attempts to change their behavior before they meet their goals and move to the next stage (Marcus et al., 1996). In moving through these stages, people can use different strategies and techniques depending on their goals and motivation to participate in PA (Lee et al., 2006).

Self-efficacy is persons’ belief in their own ability to organize and execute the course of action required to achieve given goals and resist the temptation to relapse. Self-efficacy is important because individuals with high self-efficacy for tasks tend to try harder and experience more positive emotions related to the tasks (Bandura, 1977). People with higher self-efficacy tend to accept more challenges and persist with their appointed tasks despite obstacles. Meanwhile, the pros and cons in decisional balance refer to the perceived positive and negative aspects of modifying individuals’ behavior (Janis and Mann, 1977). Individuals tend to adopt new behavior when they find that the benefits outweigh the cons. Processes of change are strategies using cognitive and behavioral processes to modify experience and environment to change behavior (Prochaska and Velicer, 1997). Cognitive and behavioral processes, which are the second-higher order model in process of changes, can be used to sustain improvements in PA.

Over the decades, many studies have demonstrated a significant relationship between the TTM and PA behavior (Kim, 2007; Kim and Cardinal, 2010; Jeon et al., 2014). The TTM can provide useful guidance for Malaysia’s Ministry of Health and Ministry of Education to develop a strategic plan to promote and create awareness of the importance of regularly engaging in PA. The agencies can work hand in hand to establish health policy by educating university students and parents and regularly conducting exercise programs. Moreover, sports medicine professionals, such as certified athletic trainers, sport psychologists, and physical therapists, can refer to TTM studies as sources to understand the need to encourage university students to participate in PA by improving their level of processes of change.

**FIGURE 1:**
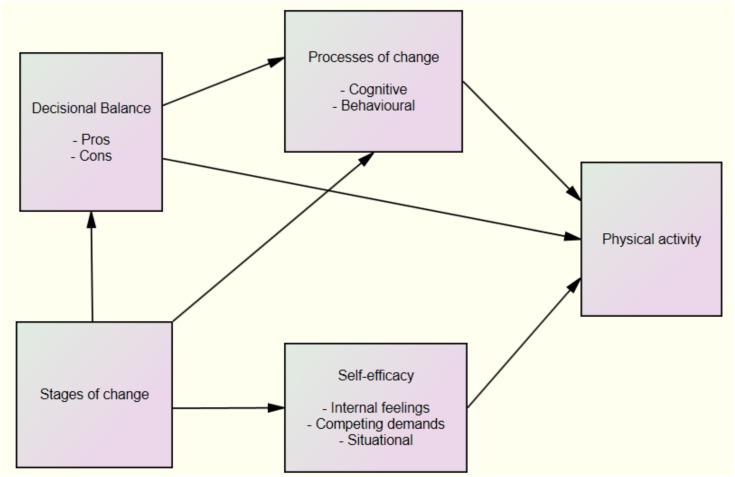
Hypothesized model for explaining undergraduate students’ physical activity behavior.

However, we found only one study to date that assesses Malaysian secondary-school students’ exercise behaviors based on the TTM (Kee et al., 2010). Although the application of the TTM seems to be convincing, there is a lack of research examining university students in the stages of exercise behavior change using the TTM. The present study, therefore, was aimed at applying the TTM to examine the structural relationships between stages of change, self-efficacy, decisional balance, processes of change, and PA among university’s students who enrolled in health and medical degree program. We chose health and medical students because they usually are busy (e.g., due to high load of studies, assignments, and clinical postings) and so lack time for PA. This study was also intended to explore the direct and indirect relationships between stages of change, self-efficacy, decisional balance, processes of change and PA among the University’s students. The hypothesized relationships proposed in our study are shown in Figure 1.

## Materials and Methods

### Participants

A total of 562 undergraduate students from the Health Campus, Universiti Sains Malaysia (118 male and 444 female) participated in this study. All the eligible students registered to the university’s co-curricular program comprised of sports, arts, and uniform subjects. Only the students who were available during the time of data collection and wanted to volunteer for the study were recruited as participants. Informed consent forms were given out before the study began. Among the 600 students who gave informed consent, 562 completed the questionnaire for a response rate of 93.7%. The Monte-Carlo simulation method for sample size determination was performed using Mplus 8. The initial, hypothesized structural equation modeling (SEM) model (derived from Figure 1) was tested in a Monte-Carlo simulation study. A sample size of 560 would achieve power of at least 81% for all the regression coefficient pathways tested in the initial hypothesized SEM model. The sample size of 562, therefore, was deemed adequate for the study.

### Measures and Materials

#### Decisional Balance Scale

The decisional balance scale was a 10-item questionnaire developed by Plotnikoff et al. (2001). Using a 5-point Likert scale, the students were asked to rate their preferences for questions (e.g., “PA would help me reduce tension or manage stress”) from “not at all confident” to “extremely confident.” The two main components of the decisional balance scale were the perceived pros (advantages or benefits of exercise) and cons (disadvantages of or barriers to exercise), which represented the positive and negative aspects of individuals’ behavioral changes. The internal consistency reliability was reported to be 0.82 for pros and 0.72 for cons (Plotnikoff et al., 2001). In the present study, the internal reliability was 0.81 for the pros and was 0.82 for the cons.

#### Processes of Change Scale

The processes of change scale was a 30-item questionnaire developed by Nigg et al. (1999). Using a 5-point Likert scale, the students were asked to rate questions (e.g., “I read articles about exercise in an attempt to learn more about it”) based on their preferences from “never” to “repeatedly.” In this study, second-order factors were used in cognitive and behavioral processes. The five main components of cognitive processes were consciousness raising, dramatic relief, environmental re-evaluation, self-re-evaluation, and social liberation. The five main components of behavioral processes were counter-conditioning, helping relationships, reinforcement management, self-liberation, and stimulus control. The second-order model with two higher factors of cognitive and behavioral processes was commonly applied in research studies. The internal consistency reliability of 0.60 to 0.90 for two higher factors has been reported (Nigg et al., 1999). In the present study, the internal reliability was 0.84 for cognitive processes and 0.87 for behavioral processes.

#### Self-Efficacy Scale

The self-efficacy scale consisted of 18 items developed by Bandura (1997). Based on a psychometric study using a Korean version of the self-efficacy scale, the scale consisted of three subscales: internal feelings, competing demands, and situational (or interpersonal; Shin et al., 2001). This validated Korean version of the self-efficacy scale has been applied in a TTM study with university students conducted by Kim (2007). The students were asked to use a 5-point Likert scale ranging from 1 (cannot do) to intermediate degrees of confidence (3 = moderately certain can do) to complete confidence (5 = certain can do). In a column labeled “confidence,” the students rated how confident they were that they could regularly perform exercise routines (three or more times a week) in various circumstances (e.g., “when I am feeling tired”). Cronbach’s coefficient alpha was 0.91 for the internal consistency of the self-efficacy scale. The 2 weeks’ test***–***retest reliability was 0.86 and was performed to measure instrument stability. In the present study, the internal reliability was 0.74 for internal feelings, 0.60 for competing demands, and 0.80 for situational.

#### Stages of Change Scale

The stages of change scale consisted of one question with five stages (Marcus et al., 1992). Stages of change were assessed using a 5-item, dichotomous scale (yes/no) related to regular exercise behavior and intentions. Individuals were categorized into one of the five stages of exercise behavior change described earlier. For example, individuals choose “I currently do not exercise, and I do not intend to start exercising in the next 6 months” if they were in the precontemplation stage. In addition, 2 weeks’ test–retest reliability measures were conducted to measure instrument stability, resulting in a kappa index of coefficient of 0.78.

#### Godin Leisure-Time Exercise Questionnaire

The Godin Leisure-Time Exercise Questionnaire, developed by Godin and Shephard (1985), consisted of two questions on frequency of weekly leisure-time activities intended to gather information on the number of times participants engaged in PA. Individuals were asked to state the number of times they engaged in at least 15-min-long exercise sessions during free time weekly and to indicate the type of exercise: strenuous, moderate, or mild exercise. The frequency score was multiplied and summed by its metabolic equivalent (MET) value: (mild × 3) + (moderate × 5) + (strenuous × 9). The participants were asked to select one of three options (often, sometimes and never/rarely) for the question “During a typical 7-day period, how often do you engage in any regular exercise long enough to work up a sweat?”. The percentage of agreement between test–retest classification was reported as 72%, and the kappa coefficient was reported as 0.40 with 95% CI of 0.21 to 0.60 (Amireault and Godin, 2015).

### Procedure

A cross-sectional study design was employed in the present study. Through purposive sampling, undergraduate students who took part in co-curricular activities were recruited as study participants. A total of 29 co-curricular courses were offered during the first semester of the 2017/2018 academic year. All the students were briefed about the study and its objective.

The study was approved by Universiti Sains Malaysia’s Human Research Ethics Committee (USM/JEPeM/17070322) and conducted in accordance with the guidelines of the International Declaration of Helsinki. During the data collection phase, a sheet with essential information on the research, including the study purpose, procedures, and potential risks and benefits, was given to all the participants to enable them to clearly understand the study. They were then informed that their participation was entirely voluntary, and they were free to withdraw from the research at any time without any loss of benefits to which they were entitled or any effect on their causes. The potential participants independently decided whether to participate in the research by giving their consent.

The participants were asked to sign a consent form if they agreed to participate. They were assured that their confidentiality and privacy would be maintained, and the data collected would be used only for research purposes unless disclosure was required by law. The participants completed and returned the questionnaire to the researcher after their co-curricular sessions. To recruit a variety of students from different co-curricular activities, the researcher approached students in sports, arts, and uniform groups. The students took approximately 25 min to complete the questionnaire.

### Data Analysis

Statistical analysis was conducted using Mplus 8. Data were expressed as the mean and standard deviation (SD) for the numerical variables, while the categorical variable was tabulated using the frequency and percentages for descriptive information. SEM was carried out to examine the structure relationship of the TTM (stages of change, self-efficacy, decisional balance, and processes of change) and PA levels. The assumption of normality was not met, so MLR estimation was used in the analysis. MLR is the maximum likelihood parameter with standard errors and a chi-square test statistic (when applicable) robust to the non-normality and non-independence of observations (Yuan and Bentler, 2000). Following the MLR estimation, the model’s fitness was evaluated by several fit indices as recommended by Hair et al. (2010): standardized root mean square residual (SRMR) of less than 0.08 with its *p*-value of less than 0.05, root mean square error of approximation (RMSEA) of less than 0.05, comparative fit index (CFI) of more than 0.95, and Tucker-Lewis index (TLI) of more than 0.95.

The initial, hypothesized SEM had one exogenous variable (stages of change) and five endogenous variables (decisional balance, processes of change, self-efficacy, and PA). All the latent variables were parceled with their respective factors. In the decisional balance scale, items DB1–DB5 were parceled to D1 (pros), while items DB6–DB10 were parceled to D2 (cons). For the processes of change scale, the second-higher order model of factors cognitive processes and behavioral processes was used in SEM analysis. In the self-efficacy scale, four items (i.e., SE2, SE5, SE6, and SE7), three items (i.e., SE4, SE8, and SE10), and five items (i.e., SE11, SE12, SE13, SE16, and SE17) were parceled to S1 (internal feelings), S2 (competing demands), and S3 (situational), respectively. PA was calculated to obtain the mean total of each participant’s MET score. The initial, hypothesized SEM was tested to detect any significant pathways (*p* < 0.05) in the model. The recommended significant paths in Mplus 8 were evaluated based on theoretical support. The final SEM was presented in pathways, standardized regression weights (β), and *p*-value. In addition, direct and indirect paths related to PA are further discussed in the following section.

## Results

### Participants

The participants had a mean age of 19.81 years (SD 1.22), with an age range of 17–27 years. The majority of the students were from the School of Health Sciences (67.3%) and engaged in approximately three exercise sessions (mean: 2.62) per week, and the average per session was 43 min long. The students in sports and art groups reported having more participants than uniform groups. The demographic characteristics of the undergraduate students are shown in Table 1. Most of the students 262 (46.6%) were in art groups, while 260 (46.3%) students were in sports groups, and only 40 (7.1%) students were in uniform groups.

**Table 1:** Demographic characteristic of undergraduate health and medical students.

Characteristic	Frequency	Percentage	Mean (*SD*)
**Gender**			
Male	118	21.0	
Female	444	79.0	
**Age (years)**			19.81 (1.22)
**Race**			
Malay	412	73.3	
Chinese	95	16.9	
Indian	33	5.9	
Others	22	3.9	
**Field of study**			
School of Medical Sciences	123	21.9	
School of Health Sciences	378	67.3	
School of Dental Sciences	61	10.9	
**Years of study**			1.51 (0.81)
**Curriculum involvement**			
Sport group	260	46.3	
Art group	262	46.6	
Uniform group	40	7.1	
Exercise frequency per week			2.62 (1.64)
Exercise period per session (min)			43.14 (41.14)

### Initial Structural Model

The results of model fit for the initial model are shown in Table 2. There were seven hypothesized path relationships in the initial model. However, the model displayed a poor fit with the data (CFI = 0.720; TLI = 0.561; SRMR = 0.114; RMSEA (CI: 90%) = 0.134 (0.119, 0.149); RMSEA *p* < 0.001). All the fit indices did not meet the recommended value, so some modifications were made by removing the non-significant paths between variables that did not explain much of the model. The most non-significant paths was removed one at a time in the model and repeatedly re-tested for fitness. However, removal of paths was based on their theoretically meaningfulness.

**Table 2:** Model Fit Indices for model of initial structural model.

Model	CFI	TLI	SRMR	RMSEA (90%CI)	RMSEA *p*-value
Model 1 (Initial)	0.720	0.561	0.114	0.134 (0.119, 0.149)	<0.001

*CFI, Comparative Fit Index; TLI, Tucker-Lewis index; SRMR, Standardized Root Mean square Residual; RMSEA, Root Mean Square Error of Approximation; CI, Confidence Interval.*

### Modified Structural Model

Table 3 shows the results of modified structural model 2 to model 4. The model fit indices showed that models 2 to model 4 did not fit the data well. Model 2 displayed the results after removing the non-significant path of pros’ affect on PA (CFI = 0.722; TLI = 0.564; SRMR = 0.114; RMSEA (CI: 90%) = 0.133 (0.119, 0.148); RMSEA *p* < 0.001). Although the fit indices were improved, the model still not display acceptable fit indices within the recommended range of values. The structural model was tested again until all the non-significant paths were removed. After several analyses, model 4 still did not display acceptable results (CFI = 0.770; TLI = 0.587; SRMR = 0.104; RMSEA (CI: 90%) = 0.130 (0.114, 0.146), RMSEA *p* < 0.001).

**Table 3:** Model Fit Indices for model of Model 2 to Model 4.

Model	CFI	TLI	SRMR	RMSEA (90%CI)	RMSEA *p*-value
Model 2^a^	0.722	0.564	0.114	0.133 (0.119, 0.148)	<0.001
Model 3^b^	0.724	0.549	0.113	0.136 (0.121, 0.151)	<0.001
Model 4^c^	0.770	0.587	0.104	0.130 (0.114, 0.146)	<0.001

*CFI, Comparative Fit Index; TLI, Tucker-Lewis index; SRMR, Standardized Root Mean square Residual; RMSEA, Root Mean Square Error of Approximation; CI, Confidence Interval. ^a^Remove nonsignificant pathway of Pros affects physical activity. ^b^Remove nonsignificant pathways of Cons affects physical activity and Cons affects processes of change. ^c^Remove nonsignificant pathway of self-efficacy affects physical activity.*

### Final Structural Model

**FIGURE 2:**
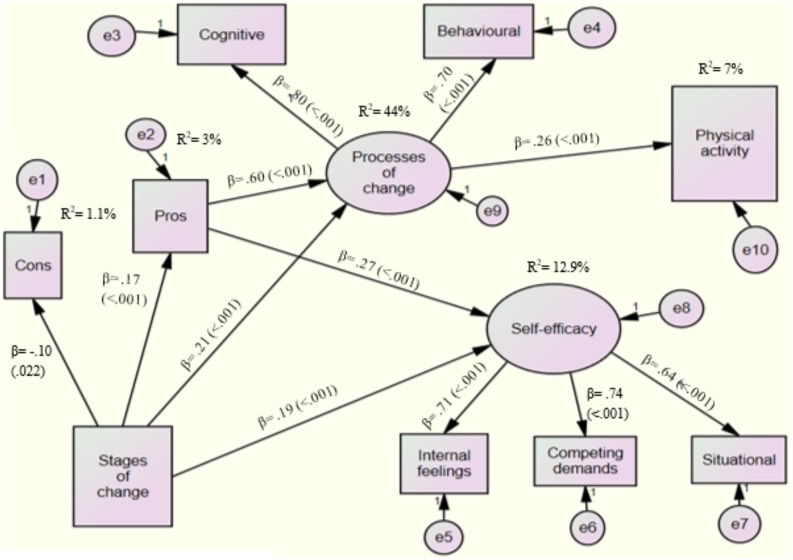
Final structural model for explaining undergraduate students’ physical activity behavior.

Table 4 shows the overall results of the final structural model (Model 5). Model 5, which was the parsimonious model, was achieved after adding new paths of pros’ affects on self-efficacy (Figure 2). The results indicated that Model 5 fit the data well, and the majority of the fit indices were within the recommended values (CFI = 0.947; TLI = 0.908; SRMR = 0.046; RMSEA (CI: 90%) = 0.061 (0.045, 0.078); RMSEA *p* = 0.130).

**Table 4:** Model Fit Indices for model of final structural model.

Model	CFI	TLI	SRMR	RMSEA (90%CI)	RMSEA *p*-value
Model 5	0.947	0.908	0.046	0.061 (0.045, 0.078)	0.130

*CFI, Comparative Fit Index; TLI, Tucker-Lewis index; SRMR, Standardized Root Mean square Residual; RMSEA, Root Mean Square Error of Approximation; CI, Confidence Interval.*

Based on the final structural model (see Figure 2), the standardized coefficients embedded in the structural model indicated that stages of change had statistically significant, direct effects on pros (*p* < 0.001), cons (*p* = 0.022), processes of change (*p* < 0.001), and self-efficacy (*p* < 0.001). Among all the variables, only processes of change had statistically significant, direct effect on PA. Of the TTM variables, pros had the strongest, direct effect on processes of change. Processes of change in the structural model explained 7% of the variance in PA. Pros and stages of change explained 44% of the variance in processes of change and 13% of the variance in self-efficacy.

### Indirect Relationships in the Final Structural Model

Although there was no significant, direct path relationship between PA and stages of change, their total indirect relationship was shown to be positive and statistically significant (β = 0.080, *p* < 0.001). The two specific indirect effects of stages of change to PA also returned statistically significant results via processes of change (β = 0.053, *p* = 0.003) and via pros and processes of change (β = 0.027, *p* = 0.002). In addition, the specific indirect effect of pros to PA via processes of change (β = 0.153, *p* < 0.001) was also statistically significant.

## Discussion

The present study provides new contributions to existing knowledge on the relationship between the TTM and PA among Malaysian health and medical undergraduate students. The final model was modified according to the conceptual framework proposed by Kim and Cardinal (2010). To obtain deeper understanding of how the TTM led to PA involvement among health and medical students, this study added the full constructs of the TTM to test Godin’s PA (Godin and Shephard, 1985).

Based on the initial structural model, this study suggests that processes of change, decisional balance, and self-efficacy can be affected by stages of change and can affect students’ PA. The initial structural model indicates that students with high self-efficacy and positive thoughts and behavior tend to be more physically active despite facing numerous obstacles (e.g., pressure from examinations, postings, medical rotations, and coping with studies). It can be seen clearly that stages of change are the main factor contributing to the cognitive and behavior processes in processes of change that affect students’ involvement in PA. In this study, Malaysian health and medical students are mostly in the preparation stage and engage in some exercise but not regularly. A probable explanation of this result is that they exercise in their leisure time or when accompanied by friends. Co-curricular activities engage many students in PA, indicating that they are more motivated to be involved in PA when in a supportive environment, such as university exercise facilities. Friends’ support also positively affects university students’ PA by providing a supportive social network that facilitates regular involvement in PA (Kim and Cardinal, 2010). Encouragement and support from friends are important for students who intend to exercise but participate irregularly. Moreover, regularly scheduled PA sessions with company can help sustain healthier, active lifestyles. Students in the preparation stage thus are likely to change their current PA level to the action stage.

The study outcomes provide new understanding of the current exercise stages of Malaysian university students in health and medicine. The findings indicate that, decisional balance, and processes of change influence continuance of regular exercise among students depending on their current stage of change. These results help us to understand that most university students in the preparation stage are likely to participate in PA if they consider its advantages and disadvantages, have positive thoughts and good behavior. Although the present study did not found significant relationship between self-efficacy and PA, Cho (2010) claimed reported that those in the preparation and action stages have higher self-efficacy than those in the pre-contemplation and contemplation stages. Increasing self-efficacy in early stages of change, therefore, can help improve students’ engagement in PA.

For decisional balance, the pros and cons have statistically significant relationships with stages of change. The perceived positive and negative aspects of PA depend on students’ current stage of exercise behavior. Students in early stages tend to perceive regularly engaging in PA as a waste of time, whereas students in later stages tend to be willing to engage in PA on a regular basis regardless of obstacles. The pros and cons are important components in continuous exercise, and active students have higher positive factors scores for doing exercise than students with low activity levels (Karaca et al., 2016). Similarly, Prochaska and Velicer (1997) reported that a positive decisional balance is associated with motivational readiness for exercise. It, therefore, is important to create a positive decisional balance in the early stages of PA (Kang and Kim, 2017).

In the present study, processes of change have the largest total effect on university students’ current stages of PA. Students’ stages of change was significantly positive associated with processes of change. In other words, current exercise level could have positive effect on their thought and behavior in engaging PA. This finding replicates previous research reporting that all processes of change (cognitive and behavioral) have correlations in all stages of change (Hwang and Kim, 2011). In addition, a study by Romain et al. (2014) supported that processes of change components are better predictors of transitions between stages of change.

Despite evidence on the effectiveness of reaching PA levels, self-efficacy and decisional balance have weak relationships with PA. Among the TTM’s constructs, only processes of change are found to affect PA in the final structural model. This finding supports the need to use cognitive processes to think of the benefits of PA and to have good behavior to engage in PA regularly. This result can be explained by students’ ability to make their own decisions about their health and the adoption of regular PA. Interestingly, this health-enhancing behavior in cognitive processes may reflect a narrow view of positive psychological outcomes (Millstein et al., 1994; Ratey and Hagerman, 2013). In the present study, students’ processes of change was significantly positive associated with PA. This indicated that those who reported to have higher level of processes of change behavior would also have higher amount of PA. The finding is similar to a U.S. study reporting that Mexican-American young women use processes of change to become more physically active (Benitez et al., 2017).

However, the present study’s results conflict with the work of Jeon et al. (2014), who found that self-efficacy is a factor contributing to PA behavior. As well, Kim and Cardinal (2010) reported that self-efficacy has a greater total effect on PA behavior than pros and cons. They found that self-efficacy is the strongest predictor of adopting and maintaining regular PA behavior (Kim and Cardinal, 2010). This result was proven by the finding that adolescents with high self-efficacy are more likely to engage in PA than adolescents with lower levels of self-efficacy (Kim and Cardinal, 2010). In addition, PA can affect individual self-efficacy in a conceptual model proposed by Elavsky et al. (2009). This hypothesis was tested in a study conducted by Joseph et al. (2014), who found that college students with higher PA levels report greater exercise self-efficacy.

This study has several limitations that need to be considered. The sample was comprised of university students who exercised during co-curricular activities, so the results have limited generalizability to other undergraduates and do not reflect all undergraduates in Malaysia. However, researchers can use the sample from this university as the reference group in future studies. A cross-cultural study using the TTM construct to observe exercise behavior can be conducted among the Malaysian broader population and in other countries.

## Conclusion

In conclusion, the present study showed that the TTM has a positive relationship with PA. To engage regularly in PA, individuals must have positive thoughts and good behavior in processes of change, which push them and help them keep motivated to engage in PA. This can be clearly seen as the students’ current stages of exercise and positive behavior and thoughts might influence them to engage in regular PA. They are more willing to engage in PA after considering the benefits of exercise. The results show that pros and cons have important impacts on the students’ current stages of exercise. The higher the stage of exercise, the more likely an individual is to select suitable exercise and to be willing to conduct PA at any time. In addition, healthy behavior interventions, such as the exercise type and the participants’ cognitive and behavior aspects, must be taken into consideration. These findings provide recent evidence that processes of change are the most crucial factor affecting PA.

## Author Contributions

KTL, GK, YCK, YK, and WNA developed the concept and design of the study. KTL and GK conducted the study, participated in data collection, and drafted the original manuscript. KTL and YCK analyzed and interpreted the data. GK, YCK, YK, and WNA provided critical revisions to the manuscript. All authors read and approved the final manuscript.

## Conflict of Interest Statement

The authors declare that the research was conducted in the absence of any commercial or financial relationships that could be construed as a potential conflict of interest.

## References

[B1] Amireault S. Godin G. (2015). The godin-shephard leisure-time physical activity questionnaire: validity evidence supporting its use for classifying healthy adults into active and insufficiently active categories. *Percept. Mot. Skills* 120 604–622. 10.2466/03.27.PMS.120v19x7 25799030

[B2] Bandura A. (1977). Self-efficacy: toward a unifying theory of behavioral change. *Psychol. Rev.* 84 191–215. 10.1037/0033-295X.84.2.191 847061

[B3] Bandura A. (1997). *Self-Efficacy: The Exercise of Control.* New York: W. H. Freeman Company.

[B4] Benitez T. J. Tasevska N. Indiana K. C. Keller C. (2017). Cultural relevance of the transtheoretical model in activity promotion: Mexican-American women’s use of the process of change. *J. Health Dispar. Res. Pract.* 10 20–27. PMC13102281 42027718

[B5] Cho S. Y. (2010). *Comparison of Processes of Change, Self-efficacy, Decisional Balance and Personality by the Stages of Exercise Behavior in Middle-Aged Women.* Master Thesis, Aju University, School of Public Health, Los Angeles, CA.

[B6] Elavsky S. McAuley E. Motl R. W. Konopack J. F. Marquez D. X. Hu L. (2009). Physical activity enhances long-term quality of life in older adults: efficacy, esteem, and affective influences. *Ann. Behav. Med.* 30 138–145. 10.1207/s15324796abm3002-6 16173910

[B7] Godin G. Shephard R. J. (1985). A simple method to assess exercise behaviour in the community. *Can. J. Appl. Sport Sci.* 10 141–146. 4053261

[B8] Hair J. F. Black W. C. Babin B. J. Anderson R. E. (2010). *Multivariate Data Analysis*, 7th Edn. Upper Saddle River, NJ: Pearson Prentice Hall.

[B9] Han H. Gabriel K. P. Kohl H. W. (2015). Evaluations of validity and reliability of a transtheoretical model for sedentary behavior among college students. *Am. J. Health Behav.* 39 601–609. 10.5993/AJHB.39.5.2 26248170

[B10] Han H. Pettee Gabriel K. Kohl H. W. III (2017). Application of the transtheoretical model to sedentary behaviors and its association with physical activity status. *PLoS One* 12:e0176330. 10.1371/journal.pone.0176330 28448531 PMC5407750

[B11] Hwang J. Kim Y. H. (2011). Adolescents’ physical activity and its related cognitive and behavioural processes. *J. Biol. Sport* 28 19–22. 10.1016/j.jsams.2017.07.006 28778824

[B12] Institute for Public Health (2014). *National Health and Morbidity Survey 2014: Malaysian Adult Nutrition Survey (MANS) Survey Findings*, Vol. 2 Kuala Lumpur: Ministry of Health Malaysia, 343.

[B13] Janis I. L. Mann L. (1977). *Decision Making: A Psychological Analysis of Conflict, Choice and Commitment.* New York, NY: Free Press.

[B14] Jeon D.-J. Kim K.-J. Heo M. (2014). Factors related to stages of exercise behavior change among university students based on the transtheoretical model. *J. Phys. Ther. Sci.* 26 1929–1932. 10.1589/jpts.26.1929 25540500 PMC4273060

[B15] Joseph R. P. Royse K. E. Benitez T. J. Pekmezi D. W. (2014). Physical activity and quality of life among university students: exploring self-efficacy, self-esteem, and affect as potential mediators. *Q. Life Res.* 23 659–667. 10.1007/s11136-013-0492-8 23928820 PMC4049193

[B16] Kang S. J. Kim Y. H. (2017). Application of the transtheoretical model to identify predictors of physical activity transition in university students. *J. Sport Psychol.* 26 6–11.

[B17] Karaca A. Caglar E. Deliceoglu G. Bilgili N. (2016). Physical activity with regard to socio-demographic variables and decisional balance perceptions for exercise among university students. *J. Phys. Educ. Sport* 16 932–939.

[B18] Kee K. M. Ong T. F. Wee E. H. (2010). *Exercise Behavior of Malaysian Secondary School Students: A Transtheoretical Model Approach.* Kuala Lumpur: Paper presented at the International Conference on Science and Social Research 10.1109/CSSR.2010.5773851

[B19] Kim Y. H. (2007). Application of the transtheoretical model to identify psychological constructs influencing exercise behaviour: a questionnaire survey. *Int. J. Nurs. Stud.* 44 936–944. 10.1016/j.ijnurstu.2006.03.008 16698024

[B20] Kim Y. H. Cardinal B. J. (2010). Psychosocial correlates of korean adolescents’ physical activity behavior. *J. Exerc. Sci. Fit.* 8 97–104. 10.1016/S1728-869X(10)60015-9

[B21] Kueh Y. C. Kuan G. Morris T. (2017). The physical activity and leisure motivation scale: a confirmatory study of the malay language version. *Int. J. Sport Exerc. Psychol.* 1–16. 10.1080/1612197X.2017.1321029

[B22] Lee Y. M. Park N. H. Kim Y. H. (2006). Process of change, decisional balance, self-efficacy and depression across the stages of change for exercise among middle aged women in Korea. *J. Korean Acad. Nurs.* 36 587–595. 10.4040/jkan.2006.36.4.587 16825842

[B23] Marcus B. H. Rakowski W. Rossi J. S. (1992). Assessing motivational readiness and decision making for exercise. *Health Psychol.* 11 257–261. 10.1037/0278-6133.11.4.257 1396494

[B24] Marcus B. H. Simkin L. R. Rossi J. S. Pinto B. M. (1996). Longitudinal shifts in employee’s stages and process of exercise behaviors change. *Am. J. Health Promot.* 10 195–200. 10.4278/0890-1171-10.3.195 10163299

[B25] Millstein S. G. Petersen A. C. Nightingale E. O. (1994). *Promoting the Health of Adolescents: New Directions for the Twenty-first Century.* New York: Oxford University Press.

[B26] Mozaffarian D. Afshin A. Benowitz N. L. Bittner V. Daniels S. R. Franch H. A. (2012). Population approaches to improve diet, physical activity, and smoking habits: a scientific statement from the American heart association. *Circulation* 126 1514–1563. 10.1161/CIR.0b013e318260a20b 22907934 PMC3881293

[B27] Nigg C. R. Norman G. J. Rossi J. S. Benisovich S. V. (1999). “Processes of exercise behavior change: redeveloping the scale,” in *Poster at the SBM*, San Diego, CA.

[B28] Plotnikoff R. C. Blanchard C. Hotz S. B. Rhodes R. (2001). Validation of the decisional balance scales in the exercise domain from the transtheoretical model: a longitudinal test. *Meas. Phys. Educ. Exerc. Sci.* 5 191–206. 10.1207/S15327841MPEE0504-01

[B29] Prochaska J. O. DiClemente C. C. (1983). Stages and processes of self-change of smoking: toward an integrative model of change. *J. Consult. Clin. Psychol.* 51 390–395. 10.1037/0022-006X.51.3.390 6863699

[B30] Prochaska J. O. Velicer W. F. (1997). The transtheoretical model of health behavior change. *Am. J. Health Promot.* 12 38–48. 10.4278/0890-1171-12.1.38 10170434

[B31] Rajappan R. Selvaganapathy K. Liew L. (2015). Physical activity level among university students: a cross-sectional survey. *Int. J. Physiother. Res.* 3 1336–1343. 10.16965/ijpr.2015.202

[B32] Ratey J. J. Hagerman E. (2013). *Spark: The Revolutionary New Science of Exercise and the Brain.* New York, NY: Little, Brown and Company.

[B33] Rhodes R. E. Nigg C. R. (2011). Advancing physical activity theory: a review and future directions. *Exerc. Sport Sci. Rev.* 39 113–119. 10.1097/JES.0b013e31821b94c8 21705861

[B34] Romain A. J. Attalin V. Sultan A. Boegner C. Gernigon C. Avignon A. (2014). Experiential or behavioral processes: which one is prominent in physical activity? Examining the processes of change 1 year after an intervention of therapeutic education among adults with obesity. *Patient Educ. Couns.* 97 261–268. 10.1016/j.pec.2014.08.004 25181999

[B35] Shin Y. H. Jang H. J. Pender N. J. (2001). Psychometric evaluation of the exercise self-efficacy scale among Korean adults with chronic diseases. *Res. Nurs. Health* 24 68–76. 10.1002/1098-240X(200102)24:1<68::AID-NUR1008>3.0.CO;2-C 11260587

[B36] U.S. Department of Health, and Human Services (2008). *Physical Activity Guidelines for Americans.* Washington, DC: Department of Health and Human Services.

[B37] U.S. Department of Health and Human Services and U.S. Department of Agriculture (2015). *2015 – 2020 Dietary Guidelines for Americans*, 8th Edn. Available at https://health.gov/dietaryguidelines/2015/guidelines/

[B38] World Health Organization (2010). *Global Recommendations on Physical Activity for Health.* Available at: http://www.who.int/dietphysicalactivity/publications/9789241599979/en/26180873

[B39] Yuan K. H. Bentler P. M. (2000). Three likelihood-based methods for mean and covariance structure analysis with nonnormal missing data. *Sociol. Methodol.* 30 165–200. 10.1111/0081-1750.00078

